# Deciphering leptospirosis eco-epidemiology in New Caledonia

**Published:** 2011-01-10

**Authors:** J Perez, F Brescia, J Becam, C Goarant

**Affiliations:** 1 Institut Pasteur de Nouvelle-Calédonie, Nouméa, New Caledonia; 2 Institut Agronomique Néo-Calédonien, Nouméa, New Caledonia

## 

Leptospirosis is a zoonosis of worldwide distribution with major incidence in the tropics. In New Caledonia, it has been studied for years and is regarded as a major public health concern. It is characterized by an endemic pattern with seasonal epidemics arising during rainy periods, notably during “la Niña” periods. Both the endemic and epidemics have a rural epidemiology and result from the infection by a variety of *Leptospira* strains. The Institute Pasteur in New Caledonia (IPNC) is the reference laboratory for human leptospirosis and has been studying this disease for years, notably developing and implementing efficient molecular diagnostic tools. At the same time, an expertise on this disease has led to numerous studies on the epidemiology and physiopathology of leptospirosis.

IPNC recently started a study aiming at identifying the *Leptospira* strains currently circulating in New Caledonia and their corresponding animal reservoirs. Because leptospirosis is a complex pathology involving reservoir hosts, environmental sources of infection and susceptible mammals including Man, the control of human leptospirosis has to take these animal reservoirs into account. In any region, a limited number of pathogenic *Leptospira* strains are circulating which can cause disease in humans. Every single *Leptospira* strain is supposed to be maintained by one or several reservoir animal host species. Current molecular methods allow identifying the infecting *Leptospira* strain at a sub-species level, possibly by directly using these molecular tools with DNA extracted from animal and human clinical specimens.

We evaluated the usefulness of the sequence polymorphism of diagnostic PCR products and used this molecular tool for studying the prevalence and identification of the different *Leptospira* strains in both rodent and non-rodent Mammals. Because rodents are known to play a pivotal role in the maintenance of major *Leptospira* strains, an ecological study of rodent dynamics and their *Leptospira* prevalence is conducted in an area of highest incidence, taking seasonal and meteorological variability into account. Preliminary results confirm their role as reservoirs for *L. interrogans* Icterohaemorragiae and *L. borgpetersenii* Ballum and highlight a joint effect of temperature and pluviometry on rodents’ populations and their *Leptospira* carriage. Additionally, the introduced deer *Cervus timorensis russa* was shown to be a reservoir for *L. borgpetersenii* Hardjo.

